# A Study in Bleaching Teeth

**Published:** 1902-09-15

**Authors:** N. S. Hoff

**Affiliations:** Ann Arbor, Mich.


### A STUDY IN BLEACHING TEETH.

BY N. S. HOFF, D.D.S., ANN ARBOR, MICH.

Read at the Third Tri-annial Tri-State Meeting at Indianapolis, June 4, 1901.

The remedies generally employed in antiseptic treatment, and which leave such undesirable results, are the essential oils, eucalyptus, cloves, cassia, wintergreen, and others; carbolic acid, creosote, iodin compounds, hydronaphthol, tannic acid, and other similar drugs used in canal treatment.

In considering the methods employed to decolor teeth stained by such drugs it will be difficult to disassociate them from other accidental agencies which are liable to be associated, and which may materially modify the treatment used. To secure effective results in the shortest time, it is important that we first determine the cause of the stain and its character. This will influence the selection of the bleaching agent or process. But when a tooth is deeply stained and the condition is of long standing, the problem becomes complex indeed. If the stain is of recent occurrence and the cause be known, a proper chemical re-agent may promptly effect the desired discoloration. In old stains the drug which caused the discoloration may long since have suffered decomposition, though its effects are still present, and perhaps in an unintelligible form demanding the most penetrating and distructive pigment solvents practicable.

In ordinary bleaching of teeth chemical re-agents are employed on two principles. One is the removal of the pigmented structure by oxidation, the other, decomposing it chemically and structurally by the process of reduction. It is questionable whether both methods may not be essentially oxidation processes. They certainly act so in bleaching organic stains. In both processes mechanical and physical detergent aids are also essential to complete results. Of bleaching agents, direct oxidizers are such as contain oxygen in loose combination which can be set free without injury to the tooth and at pleasure. The peroxids are illustrations of this class. The reduction re-agents are such as have the property of breaking up pigment compounds by chemical combinations or interchange of elements having stronger mutual affinities than those which prevail in the pigment compounds or the bleaching agent itself. The chlorin and sulfurous oxid methods are good illustrations, although in many cases these agents are supposed to act as indirect oxidizers.

The question which we propose you shall consider is, which of these processes and agents are of the most value, and of practical application in decoloring teeth stained by the class of drugs above mentioned?

It may be of advantage to study this subject from its theoretical aspect, but should we attempt to do so we are likely to become disheartened, as the problem becomes so complicated that it is practically incapable of complete solution. The exact chemical nature of many of these substances used as antiseptics is not known, or, if known, is of such a complicated nature that it is difficult to determine what the reactions may be which take place when they are brought in contact with the tooth-structure in its normal condition. It is still more difficult to determine the nature of possible reactions when there is present in the tooth-structure the chemical detritus resulting from the necrotic processes. Notwithstanding these difficulties, successful methods of bleaching teeth will never be made practicable until some definite theoretical knowledge is obtained. This may be secured by laboratory research, but if it be not an actual deduction from successful clinical experience, it must necessarily be confirmed by such experience.

In order that we may get some basis, at least, for starting an investigation of this subject, we submit a brief study which we trust may be of some interest and value. That a comparative study of the powers of the well-known bleaching agents might be made, we selected a number of teeth which had been extracted for some time and were thoroughly dried. These were then cleansed of all carious substances, the apices of the roots cut off, and the pulp-canal and chamber reamed out so that no pulp-remnants were left. They were also thoroughly cleansed externally by mechanical means, and then with hot solutions of chlorinated soda. This left them practically clean and free from all organic contamination. They were then soaked for three days in the following solutions: Full strength solution of eucalyptol, old and dark oil of cloves, old oil of cassia, Black's "1, 2, 3" mixture and aristol, iodized phenol, carbolic acid, ninety-five percent solution; old creosote, solution of iodoform in ether, alcohol solution of beta-napthol, and a glycerin solution of tannic acid.

After removing them from the stains they were dried in the sun and by a gentle heat until all moisture had been driven off. The result, as may be seen by reference to the card, was a considerable discoloration in each case, varying from old gold in one to almost a purple in another.

One of each stain was then selected and cut into eight transverse sections, beginning at the apex of the root until the crown was reached. A proof set made up of one section of each of the ten stains was reserved for comparison, and the remainder were subjected to the decolorizing powers of the following bleaching agents: Twenty-five percent pyrozone, three percent peroxid of hydrogen, sodium peroxid, benzozone, aluminum chlorid, chlorinated lime, and Kirk's sodium sulphite and boric acid mixture. These re-agents are well known, with the exception of benzozone, which is a new organic compound of the peroxid class. It is benzoyl-acetyl peroxid ($C_6H_5COO_2COCH_3$), and is intended for disinfectant and antiseptic purposes. We selected it because of its being an organic peroxid. From a limited experience with it, we are satisfied that it possesses valuable properties as a bleaching agent, especially of organic stains. We have classed these agents into two divisions,—namely, the oxidizers and the reducers.

The oxidizers will include the four peroxids,—twenty-five percent pyrozone, three percent hydrogen peroxid, sodium peroxid, and the benzozone.

Dr. Kirk, in the "American Text-Book of Operative Dentistry," states that aluminum chlorid, as used in the Harlan process, has no bleaching properties, and that peroxid of hydrogen used in the process is the only effective agent, the aluminum chlorid serving only to set free the oxygen expeditiously. While this statement as to the reaction which occurs is undoubtedly true in the main, there is a strongly increased acid condition developed in the use of this process, which would indicate a chemical as well as a physical reaction, with the probable formation of hydrochloric acid. This acid would materially influence the bleaching process by its detergent action on both the mineral and organic stains and tissues. In our experience it seems to have had some advantage over the same strength solution of peroxid alone, and we are therefore inclined to classify it with the reduction agents.

The bleaching of the ten different stainings was under as nearly similar conditions as it was practicable to devise. Each set of sections was subjected to the several respective re-agents for twenty-four hours at room temperature, about 60° F. Of course some of the re-agents became temporarily heated while being mixed in the test-tubes. After twenty-four hours they were kept at a temperature of 100° F. for one hour, and then removed from the bleaching solutions and repeatedly washed in several changes of distilled water.

They were carefully dried for several days in the sunlight, and tabulated in their respective places on the card, where the result may be studied. As might have been expected, the twenty-five percent pyrozone makes the most creditable showing as a whole,—bleaching all the stains satisfactorily except the iodin and creosote. These two stains were the most profound, and did not yield to the oxidizing influence of the pyrozone so well as they did to the alkaline sodium peroxid.

The sodium peroxid produced almost as satisfactory bleaching as the pyrozone; none of the specimens were as clear as some of the pyrozone, but all were very materially affected. The sulphuric acid used to liberate the oxygen in the process seems to have very materially affected the inorganic part of the sections. Such effects on the teeth in the mouth would necessarily contra-indicate its use. It is unquestionably a great solvent of the organic structures.

The benzozone stands third as to effectiveness, so far as the clearness of results are concerned. It produced nearly, if not quite, as satisfactory bleaching as the sodium peroxid, and although this agent gave a decided acid reaction while bleaching, the sections do not show any destructive acid effects. The acids in this substance are organic and weak, and consequently would not affect the inorganic tooth-tissues. This agent has the advantage over the pyrozone and sodium peroxid of not being readily soluble in water. It is soluble only to a fraction of one percent. Consequently it does not act promptly, but continuously, so long as any undissolved benzozone is present. When a supersaturated solution is used, as was done in this case, or when the solution is made with heat, the bleaching continues afterward, though not so intensely as with the twenty-five percent pyrozone, but for a longer period than with any of the other bleachers. We found that there were traces of it in the fourth water used to wash out the bleaching agent. This quality would indicate that this particular agent had promise of being a very effective bleacher of organic stains, because it can be used with safety and the bleaching may be done more slowly than with the other peroxids, which are so easily decomposed when brought in contact with agents which manifest an unusual affinity for oxygen.

The three percent peroxid shows comparatively slight bleaching properties, not sufficient to make it of any great value when used by this method.

The aluminum chlorid does not make a very favorable showing; it was only slightly more effective than the three percent peroxid. It produced its best work on the iodoform and the tannic acid stains. The chlorinated lime bleaching was about the same in degree as that of aluminum chlorid. It seemed to be slightly more effective in bleaching eucalyptol and iodoform stains.

The sodium sulphite and boric acid mixture was the least effective of all the bleachers used. It does not seem to have any special affinity for the essential oils, creosote or naphthol stains; the others were only moderately bleached.

It is probably true that the methods used in making these experiments do not justify the results, as they are not practicable without material modification in practice; and it may be that some of the bleachers would be more effective if used under other conditions. Again, the staining was artificially done and may have been more profound than is likely to be met in actual practice. Nevertheless, the conditions were similar in all respects for each bleacher, and the results will undoubtedly serve as a basis for comparative judgment in selecting a bleacher for the several stains.

It certainly demonstrates one fact very significantly, and that is that bleaching of this class of discolored teeth can not be done as effectively by chemical reagents which act by decomposing the stain on the substitution theory, as it can by the oxidizing re-agents or those which act at least through molecular oxygen, probably in the form of ozone, which effectively destroys the molecular integrity of the pigment or absorbs it.

We have no doubt this conclusion will be supported also by clinical evidence. It certainly simplifies the question of selecting a proper method, as it in great measure eliminates all efforts to explain all bleaching processes on the theory of atomic disintegration. Should further research prove that the peroxids are especially adapted to bleaching this class of stains, it will simplify technical details.

Should the pigment be most easily decomposed by an organic peroxid, benzozone may be used in such a way as shall best utilize its peculiarities. Should it be a mixed or inorganic compound, the pyrozone bleacher should produce the most effective results. Should it be a pigment more soluble in the alkalies, sodium peroxid will be most satisfactory.

The principle of most expeditiously utilizing the various peroxid preparations and the divising of such methods as shall most effectively utilize all the virtues practicable of each drug or class may be left to the ingenuity of the practitioner.—*Cosmos*.

### DISCUSSION.

Dr. J. S. Cassidy: As might have been expected, Dr. Hoff's paper has been a success. It is one of the best I ever heard on the subject, a subject which is well worthy of our careful study. I am sorry that my time is too limited to discuss it as it deserves.

One fact is proven by Dr. Hoff's experiments, namely, that no one bleaching agent will answer the purpose equally well in all cases of bleaching teeth, because of the fact that the objectionable colors are due to so many different causes, even in the same tooth. Theoretically, I should presume that twenty-five percent pyrozone would be the best bleacher for the essential oils and the camphors, not alone because it is rich in oxygen, but also because the menstruum is ether, and all of the essential oils and most of their camphors are soluble in that liquid. The advantage in the sodium peroxid, as indicated in some of the specimens shown, over the pyrozone, might be due to the fact that the sulphuric acid afterward used might have acted somewhat as a bleacher itself; or because of the possibility of an oily residue in the coloring matter which would become saponified by the caustic soda, forming a soluble substance which would allow the oxygen of the peroxid to act more freely.

I want to say a word about this new drug, "benzozone." Dr. Hoff is too modest to claim the discovery himself, but to him all the credit is due of discovering this drug as a bleacher. It was introduced by the manufacturer as a germicide and disinfectant, and it is a good one. I should judge by its formula that it is rather slow in its action, that from each molecule but one atom of oxygen will act, and as Dr. Hoff suggests, this slowness of action might be an advantage, as it permits of leaving the benzozone in the tooth over night, or even longer, without injury. The acidity present is not objectionable, because it is made up mostly of benzoic and acetic acids, both of which are practically inert upon tooth-substance. So far as the chlorin compounds are concerned, I have not had much use for them as bleachers for some time. I noticed a disintegration of the enamel by the hydrochloric acid which they developed.

Now, just a word in regard to the reducing and oxidizing bodies. Both chlorin compounds and oxygen compounds might act as reducing agents under certain circumstances. The general action of chlorin itself, or chlorin compounds, on organic compounds is to remove an atom of hydrogen, an atom of chlorin taking its place. If chlorin would reduce the coloring matter in that way, the coloring matter would be destroyed in most cases, but at the same time other atoms of chlorin would reduce the water that is present, for moisture is a necessity in this process. Thus free oxygen would be given off from the moisture, and would form, in nearly all such cases, oxids that are destitute of color. Oxygen also acts in many cases as a reducing agent. It destroys alcohols by reducing them to aldehyds, and adds itself to the aldehyds to form acids, so that it may act as a reducing agent on the alcohols, but as a rule it acts directly, more easily, at a lower temperature, and in less time, than where it acts as a reducing agent. It is able to add itself to certain compounds because it can enter without disturbing the valency of the other elements in the already saturated compound; owing to its valency of 2, it is able to neutralize one unit, while introducing another.

Dr. Jno. P. Buckley, Chicago, Ills.: The subject of bleaching teeth has been somewhat slighted in the recent past by the various writers and thinkers of our profession, and it pleases me greatly to know, therefore, that the essayist has given some of his time and thought to the solving of some of the knotty problems connected with the process of bleaching teeth. The essayist has gone into an experimental study of this question. Such experimental work as this can only be spoken of in the highest terms. But, valuable though we all recognize it to be from a scientific standpoint, I do not see, in so far as the practical application of it is concerned, that it has added much to the enlightenment of the practical methods of bleaching teeth in the mouths of patients. In fact, the essayist admits this in the closing of the essay. It may be that the method used in this experimental work would not justify the results and could not be employed in practical work—that changes would have to be made before they could be made practical. Indeed, I am afraid we should have to modify them so much that in the practical work of bleaching teeth in the mouths of patients we could scarcely recognize any remnants of the methods which he has adopted.

I agree with the essayist when he says that in studying this question from the theoretical aspect we are confronted by complicated problems, but I do not believe we ought to allow these complicated problems to discourage or dishearten us. Instead of discouraging us, complicated though we recognize them to be, they ought to act as a stimulus to force us deeper into the subject that we may ultimately arrive at the correct solution.

The essayist also says, "It is still more difficult to determine the nature of possible reactions when there is present in the tooth-structure the chemical detritus resulting from the necrotic processes."

This chemical detritus which results from the decomposition of the pulp, and which is in the tooth-structure, to my mind has more to do, if not all to do, with the discoloration of the teeth that we are called upon to bleach in the mouths ot patients than have the so-called chemical or remedial agents used in the antiseptic treatment of these teeth.

The essayist has mentioned many chemicals among the class of agents which he says are ordinarily used in the antiseptic treatment of teeth. He uses oil of cassia, iodized phenol or the iodin compounds, naphthol, and some others. I had supposed that those agents had long ago been discarded by the more progressive dentists in the treatment of anterior teeth the crowns of which they expect to save in the natural condition. Such other agents as he has mentioned I do not believe stain the teeth when properly used in the antiseptic treatment of those teeth. Among those I should draw a line under eucalyptol and oil of cloves.

I want to read one more statement from this essay, and I read it in the exact words of the essayist, because I want to agree with every word in the statement. Referring to these complicated questions which are liable to discourage us, he says: "Notwithstanding these difficulties, successful methods of bleaching teeth will never be made practicable until some definite theoretical knowledge is obtained." This, to my mind, is the main thing in connection with this subject—the study of the theoretical aspect, that definite theoretical knowledge which we must have, and which we must thoroughly understand, before we can adopt any successful method of bleaching.

The essayist has gone very elaborately into the discussion of chemicals to be employed in bleaching teeth. The question of chemicals is a secondary consideration. Before we can select a proper chemical to bleach a tooth we must know definitely and specifically the nature and character of the pigment that is formed, that has discolored the tooth, before we can expect that chemical to destroy or remove that pigment.

I agree with the essayist in his discussion of the different methods and the different chemical principles employed in the bleaching of teeth. He has mentioned the oxidizers, direct and indirect, the reducing agents or the deoxidizing agents. I agree with him when he says that the best methods of bleaching these teeth is by using the oxidizing agents, those which give off their oxygen readily. In this class he has placed twenty-five percent pyrozone, sodium dioxid, the new chemical which he has referred to, benzoyl acetyl dioxid, and hydrogen dioxid. I wish, by the way, we could always be accurate in giving these agents their chemical names; the essayist has referred to them as the "peroxid," the correct chemical name being the "dioxid" of these positive radicals, whatever they may be.

In the conclusion of this essay the essayist said that naturally he found that the twenty-five percent pyrozone gave the best results in the bleaching of these stains. Sodium dioxid came second. In fact, he says that the two stains which were the most profound, the iodin and creosote, were best bleached by the alkaline sodium dioxid. All of them were nearly as well bleached by the use of this chemical as were those which were very materially affected by the twenty-five percent pyrozone.

In the practical art of bleaching teeth in the mouths of individuals, from my own limited experience I should place sodium dioxid as a bleaching agent second to none, placing the twenty-five percent pyrozone second.

Benzoyl acetyl dioxid I can only speak of from a theoretical standpoint, as I never heard of it until I saw it referred to by the essayist in his article.

Sodium dioxid is of absolutely no use to us as a bleaching agent unless we really get *sodium dioxid.* The thing that we buy ordinarily in the drug stores labeled "sodium dioxid" is nothing more nor less than sodium hydroxid. But I am supposing that we get the pure sodium dioxid, which is yellow. If this chemical is placed in the cavity of the tooth and moistened with distilled water a reaction is at once set up. Oxygen is generated, and oxygen in that allotropic form, ozone, the most active oxydizing agent we have. The by-product produced by this chemical when it is decomposed by distilled water is sodium hydroxid. The ozonized oxygen will enter the tubuli and change these iron compounds that have discolored the tooth, but does not change them into a soluble form. It changes them into another compound which is still insoluble; but the oxygen is followed up by sodium hydroxid, forming a soluble soap of the fatty globules in the tubuli, which can be washed out by simply washing the tooth in warm distilled water, mechanically bringing with it the insoluble, oxidized coloring material, and that I believe to be the way we must remove this pigment, in a detergent way. The only chemical that will aid us in doing this is sodium dioxid, the by-product being a caustic alkali.

Concerning this new chemical, "benzoyl acetyl dioxid." I see from the label on the phial that the formula is $C_6H_5COO_2COCH_3$. Under ordinary conditions I can see that it would, perhaps, by the heat of the body, unite with a molecule of water and form a molecule of benzoic acid, the formula of which is $C_6H_5COOH$, leaving an atom of nascent oxygen ($C_6H_5COO_2COCH_3 + H_2O = C_6H_5COOH + CH_3COOH + O$ nascent). Nascent oxygen is an oxidizer and hence it is a bleacher. This compound, then, undoubtedly—and I am only speaking of it from the theoretical aspect—has some bleaching properties; but the benzoic and acetic acids produced by the use of the substance, organic though they be in their nature, would not have that saponifying action upon the contents of the tubuli that we get by the use of sodium dioxid.

